# Ultrarapid Measurement of Diagnostic Antibodies by Magnetic Capture of Immune Complexes

**DOI:** 10.1038/s41598-017-03786-7

**Published:** 2017-06-19

**Authors:** Peter D. Burbelo, Sreenivasulu Gunti, Jason M. Keller, Caryn G. Morse, Steven G. Deeks, Michail S. Lionakis, Amit Kapoor, Qingxue Li, Jeffrey I. Cohen, Abner L. Notkins, Ilias Alevizos

**Affiliations:** 1 Dental Clinical Research Core, NIDCR, NIH, Bethesda, MD United States; 2 Experimental Medicine Section, NIDCR, NIH, Bethesda, MD United States; 3 Lab. of Sensory Biology, NIDCR, NIH, Bethesda, MD United States; 4 NIH Clinical Center, Critical Care Medicine Department, AIDS Section, Bethesda, MD United States; 5 Department of Medicine, University of California, San Francisco, CA United States; 6 Fungal Pathogenesis Unit, Lab. of Clinical Infectious Diseases, NIAID, Bethesda, MD United States; 7 The Research Institute at Nationwide Children’s Hospital, Columbus, Ohio United States; 8 Medical Virology Section, Lab. of Infectious Disease, NIAID, NIH, Bethesda, MD United States; 9 Sjögren’s Syndrome and Salivary Gland Dysfunction Unit, NIDCR, NIH, Bethesda, MD United States; 10 Radboud University, Nijmegen, Netherlands

## Abstract

Rapid point-of-care, antibody-based testing is not currently available for the diagnosis of most autoimmune and infectious diseases. Here we report a simple, robust and ultrafast fluid-phase immunocapture method for clinical measurements of antibody levels. This method employs neodymium magnetic sticks that capture protein A/G-coated paramagnetic beads bound to antibody-luciferase-labeled antigen complexes. We demonstrate the ability to effectively measure specific antibody levels in serum samples from patients with varied infectious or autoimmune disorders, and in the case of Sjögren’s syndrome directly in saliva, requiring about a minute per assay. We also show the feasibility of coupling this method with a hand-held luminometer for portable testing. Our method offers the potential to quickly diagnose a multitude of autoimmune and infectious diseases in point-of-care settings.

## Introduction

Point-of-care testing (POCT) is laboratory testing at or near the site of patient care^1–3^. POCT is aimed at bringing immediate test results to the patient, physician, and care team, thereby increasing the likelihood that immediate clinical management decisions can be made. Although many immunoassays have been described for detecting clinically relevant antibodies, including ELISAs and new technologies employing nanoplasmonic biosensors or electrochemical detection^4–6^, these methods are impractical for POCT due to prolonged assay times and complicated procedures. Currently, solid-phase lateral flow immunoassays and miniaturized ELISAs have been developed for POCT. However, only a few clinical tests exist using these POCT formats and require anywhere from 10 min to several hours to complete, and often yield non-quantitative results^7–9^. New technologies are needed to expand rapid and affordable POCT, especially for the diagnosis of infectious diseases associated with major public health concerns including HIV, HCV, Zika virus, or Ebola^10^, and to identify many common autoimmune diseases, for which clinicians want accurate diagnostic results as quickly as possible.

Although solid-phase immunoassays are commonly used for detecting diagnostic antibodies, fluid-phase immunoprecipitation assays often show the highest sensitivity and specificity for assessing the levels of antibodies^11^. This is because fluid-phase immunoassays efficiently maintain the discontinuous epitopes of native antigens formed by two or more non-contiguous peptide chains brought together by protein folding^11, 12^. Fluid-phase immunoassays also demonstrate a wider dynamic range for detection and can utilize shorter incubation times due to reduced diffusion lengths^13^, potentially making them ideal for POCT. A major drawback of most fluid-phase immunoassays is that they require radioactivity, which is not practical for POCT due to isotope safety and expensive instrumentation. However, one fluid-phase immunoassay technology, Luciferase Immunoprecipitation Systems (LIPS), utilizes light-emitting luciferase-antigen fusion proteins as bait in immunoprecipitation assays for measuring levels of antibodies^14^. LIPS has demonstrated high sensitivity and specificity for detecting antibodies associated with many different autoimmune and infectious diseases and has several advantages over other methods, including high signal-to-noise ratio, modular format, and the ability to multiplex^14^. LIPS immunoassays can also be processed in different ways including with a tube assay^15^, microtiter plates^16^, and a microfluidic platform^17^. Despite these many formats, LIPS usually requires >2 hours to complete an assay and involves multiple liquid handling steps, making it impractical for POCT.

In the following paper, we describe a significantly improved, streamlined modification of LIPS that is suitable for POCT. By combining light-emitting antigens with the direct capture of immune complexes on the end of a neodymium magnet, we have created an immunoassay that allows for ultrafast processing and detection of clinically relevant antibodies in one minute. Using serum or saliva, our method demonstrated high sensitivity, specificity and reliability for the diagnosis of three infectious and three autoimmune diseases, where we show robust separation between the seropositive and seronegative samples. Finally, we provide proof-of-principle that these rapid assays can be coupled with a hand-held luminometer for portable antibody detection. This highly adaptable method offers the potential to quickly diagnose a multitude of autoimmune and infectious diseases in point-of-care settings.

## Results

### Development and assay parameters of LIPSTICKS

**Figure 1:**
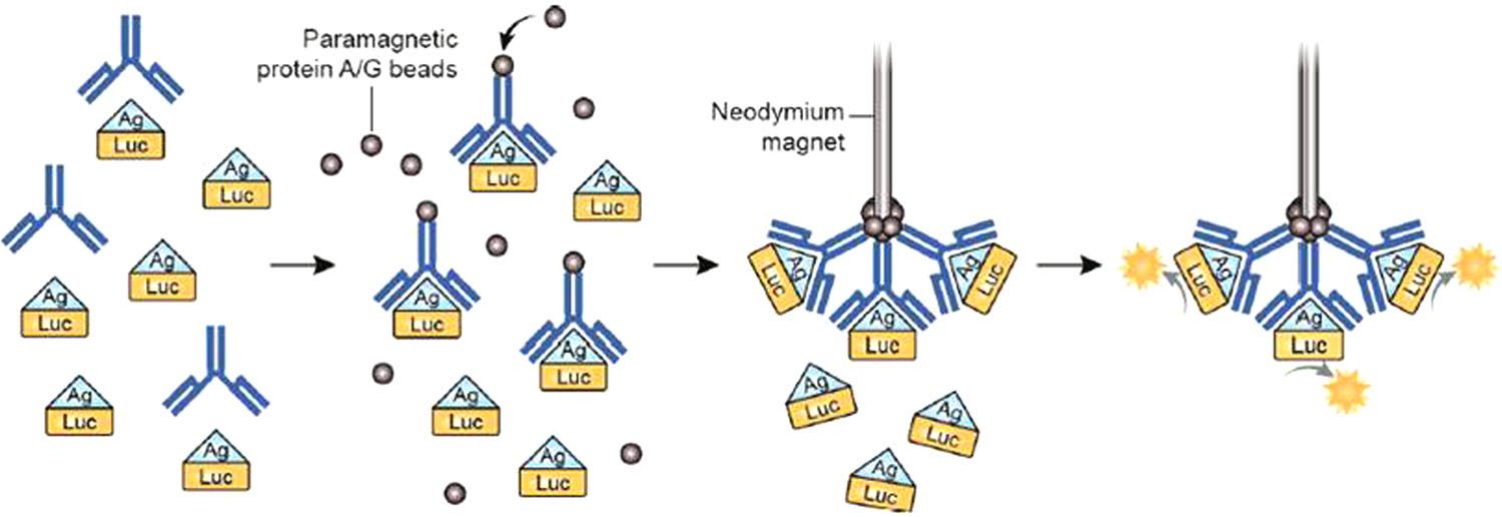
Overview of the LIPSTICKS assay. Only four simple steps are required for the 1 min antibody detection by LIPSTICKS: First, recombinant luciferase antigen fusion proteins are mixed together with diluted sera or saliva; second, paramagnetic protein A/G-coated beads are added, which bind antibody-antigen complexes; third, neodymium magnets collect the paramagnetic protein A/G-immune complexes and are briefly washed; and lastly, the magnet-bound immune complexes are placed into tubes containing luciferase substrate and emitted light is measured with a tube or hand-held luminometer.

Due to the high diagnostic performance of LIPS, we envisioned a rapid format based on a dip stick-like approach. To this end, we developed a method, termed “LIPSTICKS”, that uses magnetic “sticks” to directly capture luciferase-tagged immune complexes for the ultrafast measurement of antibodies in clinical samples (Fig. 1 and Supplemental Video 1). In LIPSTICKS, extracts of recombinant luciferase-antigen fusion proteins are incubated with serum or saliva in a microfuge tube, followed by the addition of buffer and then paramagnetic protein A/G-coated beads. The axial end of neodymium magnetic sticks are then placed directly into the reaction mixture capturing the immune complexes in seconds. This is followed by simply dipping the sticks twice in wash buffer to remove non-specifically bound antigens. The antigen-specific antibody is measured by placing the sticks into tubes containing coelenterazine or other luciferase substrates preloaded in a luminometer, where the amount of luciferase activity present is quantified in light units (LU).

With the aid of a tube luminometer, the optimum size of the magnet sticks and paramagnetic beads used in the assay was first assessed. For these experiments, extract containing a *Renilla* luciferase-HIV p24 antigen fusion protein^18^ was tested with two representative serum samples from an HIV-infected and uninfected subject. Evaluation of two different sizes of neodymium magnetic sticks of 1.59 mm and 3.18 mm diameter demonstrated that the smaller diameter magnet of 1.59 mm produced a larger LU signal because they allowed more of the light generated from the luciferase reaction to reach the photomultiplier tube. Comparison of three different sizes of protein A/G beads of 500 nm, 1 µm and 2.5 µm diameter demonstrated that all the beads had similar immunoglobulin-binding capacities, yet the 2.5 µm diameter beads were chosen because they required only a short capture time of less than 5 sec for collection from the assay solution (Supplemental Table 1).

**Figure 2:**
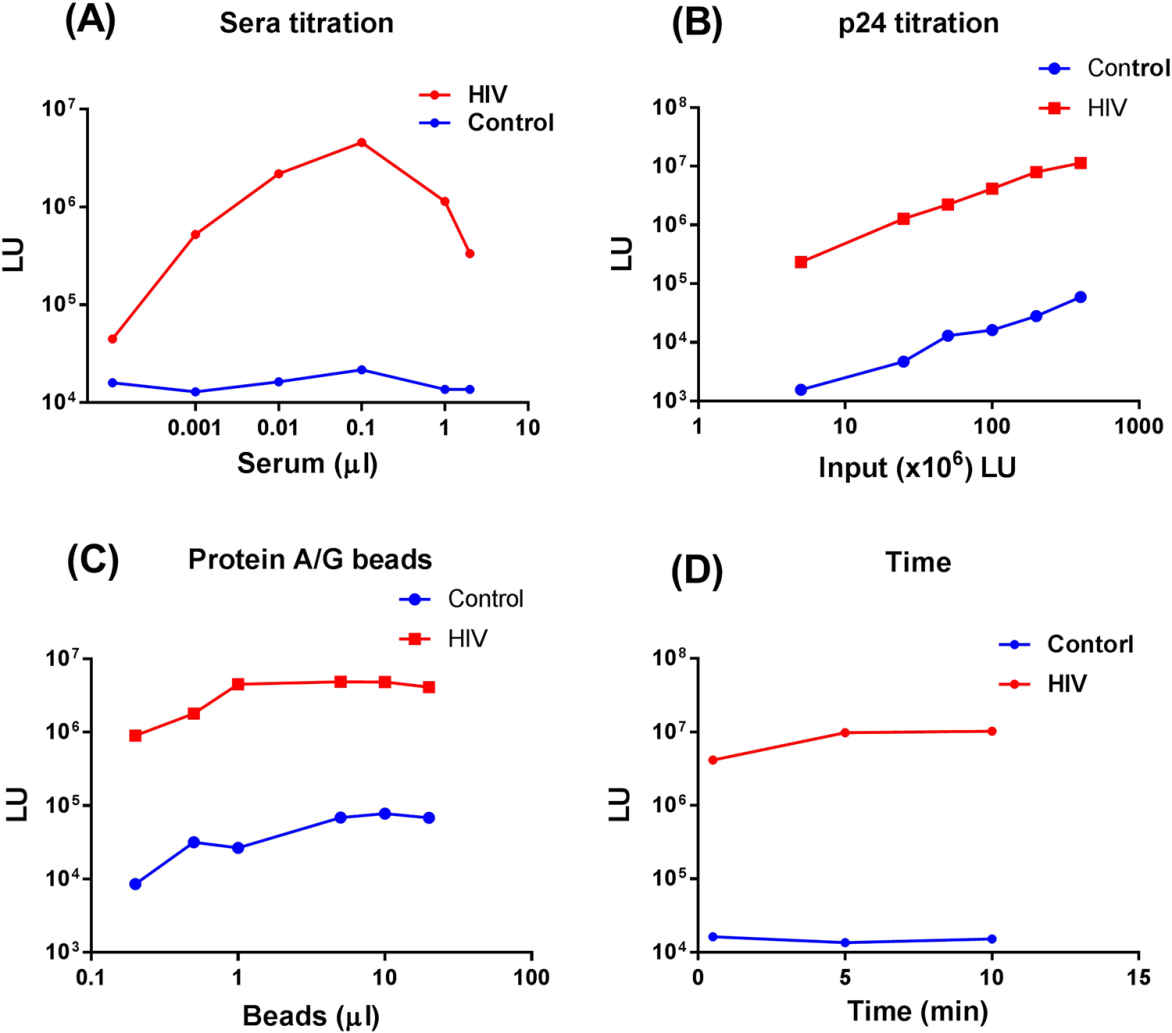
Parameters of the LIPSTICKS assay. The parameters of the LIPSTICKS assay were evaluated with one HIV negative and one HIV positive sera and is representative of multiple independent experiments. (**A**) Antibody detection by LIPSTICKS using extract containing HIV p24-luciferase fusion protein and dilutions of serum from the HIV negative (blue) and HIV positive individual (red). As shown, the highest signal to noise ratio for the HIV positive sera was obtained with 0.1 µl of sera. (**B**) Increasing the amount of HIV p24 luciferase fusion protein produces a linear increase in LU for both the HIV negative and HIV positive samples. (**C**) Increasing the amount of paramagnetic protein A/G beads produced a saturated signal at approximately 1 µl equivalent volume. (**D**) Increasing the incubation time of the sera with the HIV p24-luciferase fusion protein, from 30 seconds to 10 minutes, produced only a small increase in LU output.

Different assay conditions were next evaluated with LIPSTICKS. Varying the amount of serum from 0.001–4 µL with a fixed amount of *Renilla* luciferase-p24 antigen extract produced 24–200 times more LU from the HIV-positive compared to the HIV-negative sample (Fig. 2A). The highest signal-to-noise ratio was detected with 0.1 µL serum, yielding 21,740 LU for the HIV-negative *vs*. 4,545,000 LU for the HIV-positive sample demonstrating robust differences between the positive and negative signals given their magnitudes. Using more than 0.1 µL serum decreased the light signal due to uncomplexed immunoglobulins competing with and displacing the luciferase-tagged antigen-antibody complexes from the protein A/G beads. Titrating different amounts of *Renilla* luciferase-p24 protein extract demonstrated a roughly parallel increase in the LU in both the seronegative and seropositive samples with increasing amounts of light-emitting antigen (Fig. 2B). Similarly, varying the amount of protein A/G beads over a range of 0.1–5 µL also yielded linearity in the LU signal from both the negative and positive samples, but 1 µL beads gave a sufficiently high signal-to-noise ratio (Fig. 2C). Lastly, extending the incubation time from 1 to 5 minutes generally produced a 2-fold increase in the signal-to-noise ratio confirming the relative stability of the antibody values (Fig. 2D). These results generated under different assay conditions highlight the relative robustness of the LIPSTICKS approach, in which the relative LU detected with the seropositive sample was always well differentiated from the seronegative sample.

### Ultrarapid diagnosis of HIV by LIPSTICKS

**Figure 3:**
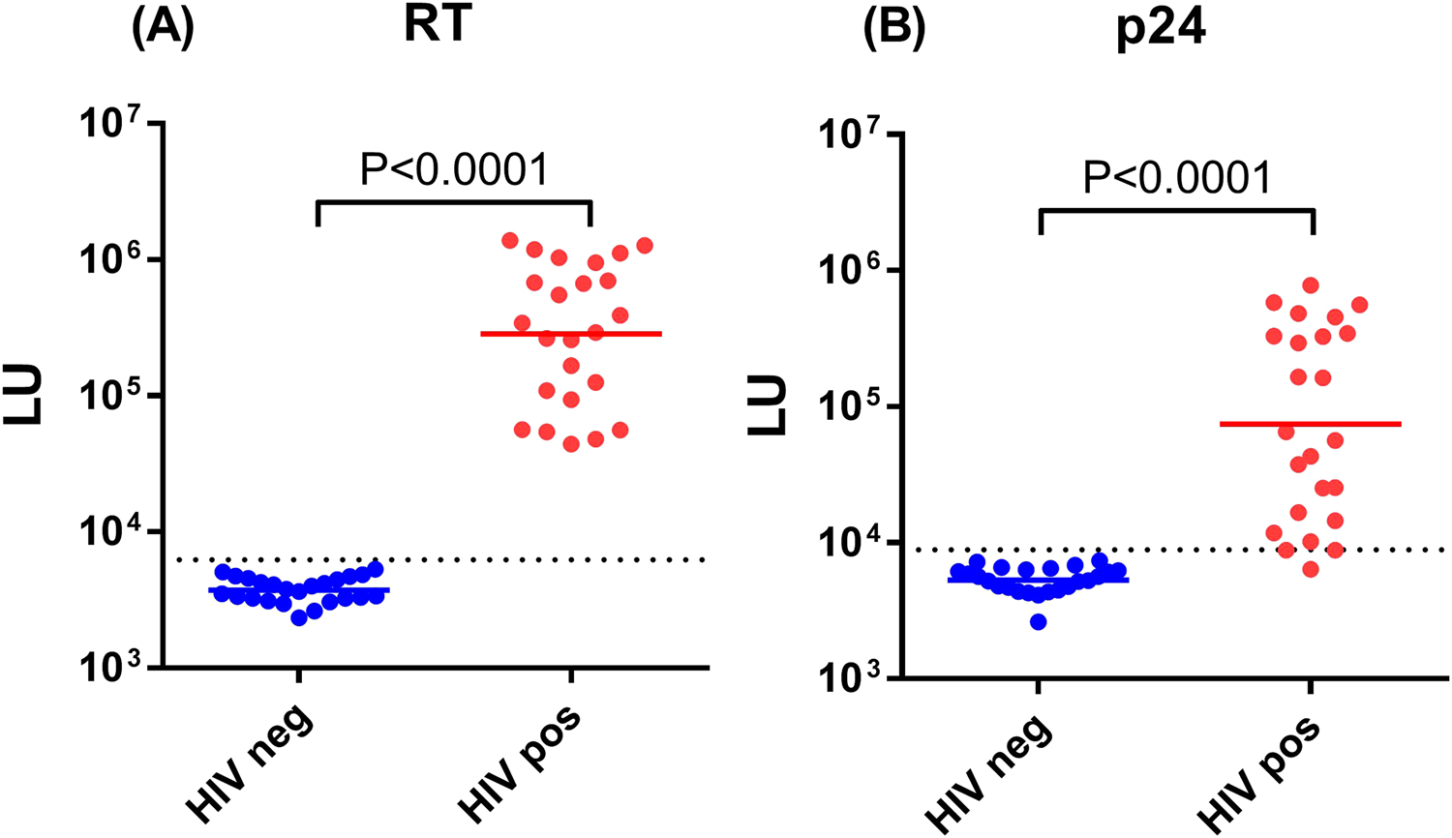
Ultrarapid Diagnosis of HIV. (**A**) HIV reverse transcriptase luciferase protein extract was used for LIPSTICKS antibody measurements with a cohort of HIV negative (blue) and HIV positive individuals (red). Each dot represent an individual subject and the geometric mean of the antibody levels in each group is shown by the colored horizontal bar. Based on the cut-off value (denote by the dotted line) for determining seropositivity, the performance of the test shows 100% sensitivity and 100% specificity for HIV diagnosis. *P* values were calculated using the Mann-Whitney *U* test. (**B**) Testing the same cohort of HIV negative (blue) and HIV positive individuals (red) with an extract of HIV p24 luciferase fusion protein demonstrated 96% sensitivity and 100% specificity. *P* values were calculated using the Mann-Whitney *U* test.

To assess the performance of the LIPSTICKS assay for antibody-based diagnosis, a cohort of uninfected (n = 24) and HIV-infected serum samples (n = 24) was evaluated for the detection of HIV infection. For this testing, extracts of *Renilla* luciferase-HIV reverse transcriptase (RT) fusion protein, known to have high diagnostic sensitivity and specificity in LIPS for HIV diagnosis^18^, was employed in LIPSTICKS under the optimized conditions as described above and in the material and methods. For this and other LIPSTICKS tests, 100 million LU of the luciferase-antigen extract was used as input per assay. As shown in Fig. 3A, the 45 sec LIPSTICKS assays generated antibody data that robustly differentiated the HIV infected from HIV-uninfected samples. The geometric mean level of anti-RT antibodies in the HIV-infected samples was 282,400 LU (95% CI 171,800–464,100) and was 76-fold higher than the uninfected controls with a value of 3,742 LU (95% CI 3417–4099). The antibody values produced by this rapid testing format were also highly reproducible with a coefficient of variation equal to 16 ± 3%. Using the mean plus three standard deviations of the uninfected controls as a cutoff value demonstrated 100% (28/28) sensitivity and 100% specificity for HIV diagnosis. Analysis of HIV antibodies in this same cohort with luciferase-p24 HIV capsid fusion protein, an antigen known to have lower diagnostic sensitivity than HIV RT^18^, demonstrated 96% sensitivity and 100% specificity (Fig. 3B). Finally, LIPSTICKS analysis of an independent set of HIV patient serum samples collected from before and after long-term antiretroviral therapy showed a statistically significant drop in anti-RT antibody levels (Supplemental Fig. 1). Since these treatment-associated decreases in antibody levels were not observed with these same samples using the standard LIPS format^18^, it is likely that the rapid, non-equilibrium, LIPSTICKS format detects antibody affinity differences and may be useful for monitoring HIV treatment. These results demonstrate that LIPSTICKS format has excellent performance for HIV diagnosis and may have applications for monitoring infection.

### LIPSTICKS diagnosis of other infectious agents

**Figure 4:**
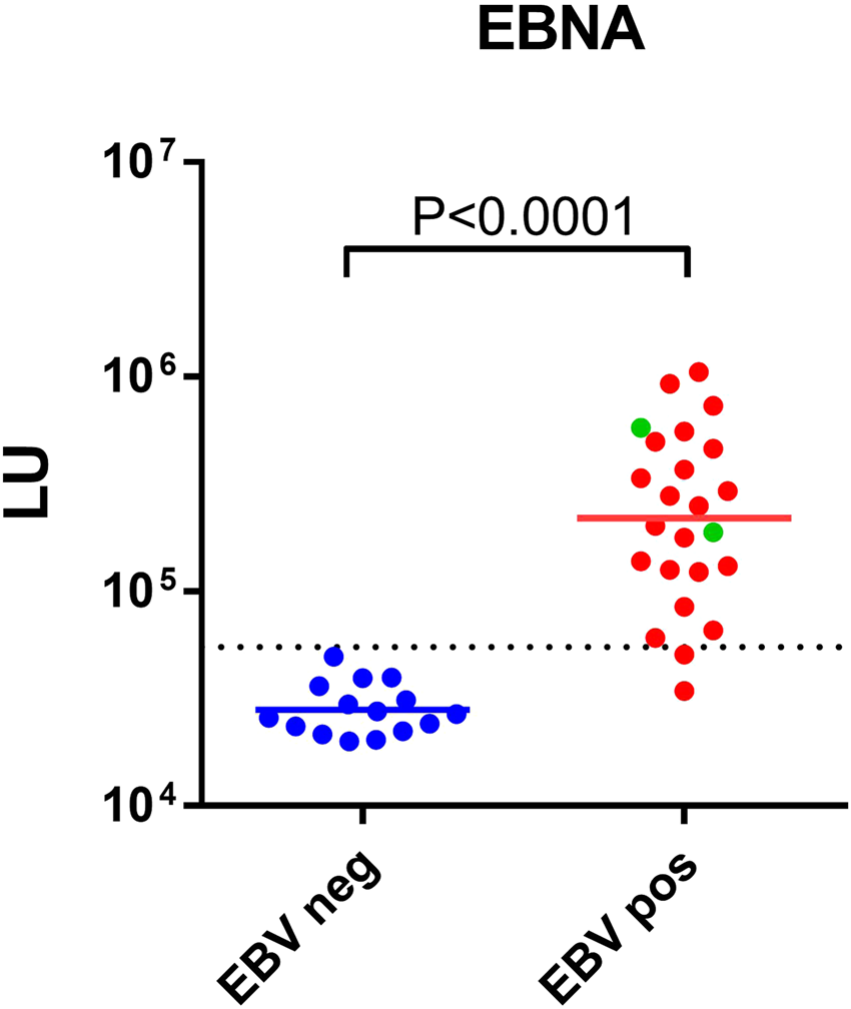
Diagnosis of EBV infection by LIPSTICKS. A cohort of human samples characterized for EBV infection by a commercial ELISA detecting antibodies directed against the capsid protein (VCA) was employed for LIPSTICKS testing. An EBV EBNA luciferase antigen in ultrarapid format showed general concordance with the ELISA for detecting EBV negative (blue) and EBV positive (red) individuals. However, two samples positive (green) by LIPSTICKS, but seronegative by the ELISA were further studied and confirmed to be true-EBV seropositive samples. The geometric mean (solid colored line) and the cut-off value (dotted line) are shown. *P* values were calculated using the Mann-Whitney *U* test.

Analysis of humoral responses is critical for the diagnosis of many human infectious diseases and for the development and monitoring of vaccines^13^. To determine the feasibility of LIPSTICKS for ultrarapid diagnosis of another human infectious disease, we tested its performance for the serological detection of Epstein-Barr virus (EBV) infection. Here, a *Renilla* luciferase-EBNA fusion protein^19^ was employed with a cohort (n = 39) of seronegative and seropositive EBV human serum samples. For serological comparison, the sensitivity and specificity of the LIPSTICKS assay were compared with the results of commercial VCA EBV ELISA. As shown in Fig. 4, the EBNA LIPSTICKS assay showed high concordance with the serological status determined by the VCA ELISA. However, four samples showed discordant results possibly related to the target antigens used in the two assays. Specifically, two ELISA seropositive samples were not detected by LIPSTICKS, but two other ELISA negative samples were seropositive by LIPSTICKS (Fig. 4). Due to the known heterogeneity in human antibody responses against EBV^19^, the two serum samples that were LIPSTICKS positive/ELISA negative were studied further. Additional analysis of the two samples confirmed seropositivity with other EBV antigens (Supplemental Table 2) and suggests that the samples represent true EBV-infected subjects and we took this into account when calculating sensitivity and specificity. Using a cut-off value derived from the mean plus three standard deviations of the EBV-uninfected controls demonstrated that the 45 sec assay yielded 92% sensitivity and 100% specificity (Fig. 4) and produced essentially identical diagnostic performance to the ELISA, which requires several hours to complete. Lastly, examination of the global index of diagnostic accuracy of this EBV LIPSTICKS test using AUC analysis revealed near perfect performance (AUC = 0.99; *P* < 0.0001).

Besides humans, there is also a need for POCT for the diagnosis of infections in animals^20^. To validate the utility of LIPSTICKS for a veterinary application, horse serum antibodies directed against the nonprimate hepacivirus virus (NPHV), a hepatitis C-like viral infection in equines^21, 22^, were examined in a small cohort of known uninfected and infected horse serum samples. As shown in Supplemental Fig. 2, a LIPSTICKS assay employing a *Renilla* luciferase-NPHV helicase antigen fusion protein detected several NPHV seropositive horse serum samples and demonstrated 86% sensitivity and 100% specificity compared to the standard LIPS assay^21^. Taken together, these promising diagnostic findings for three different infectious agents (HIV, EBV and NPHV), suggest that the LIPSTICKS technology is likely to show promise for the ultrarapid detection of antibodies associated with other human and animal infectious diseases.

### LIPSTICKS for detecting autoantibodies associated with autoimmune diseases

**Figure 5:**
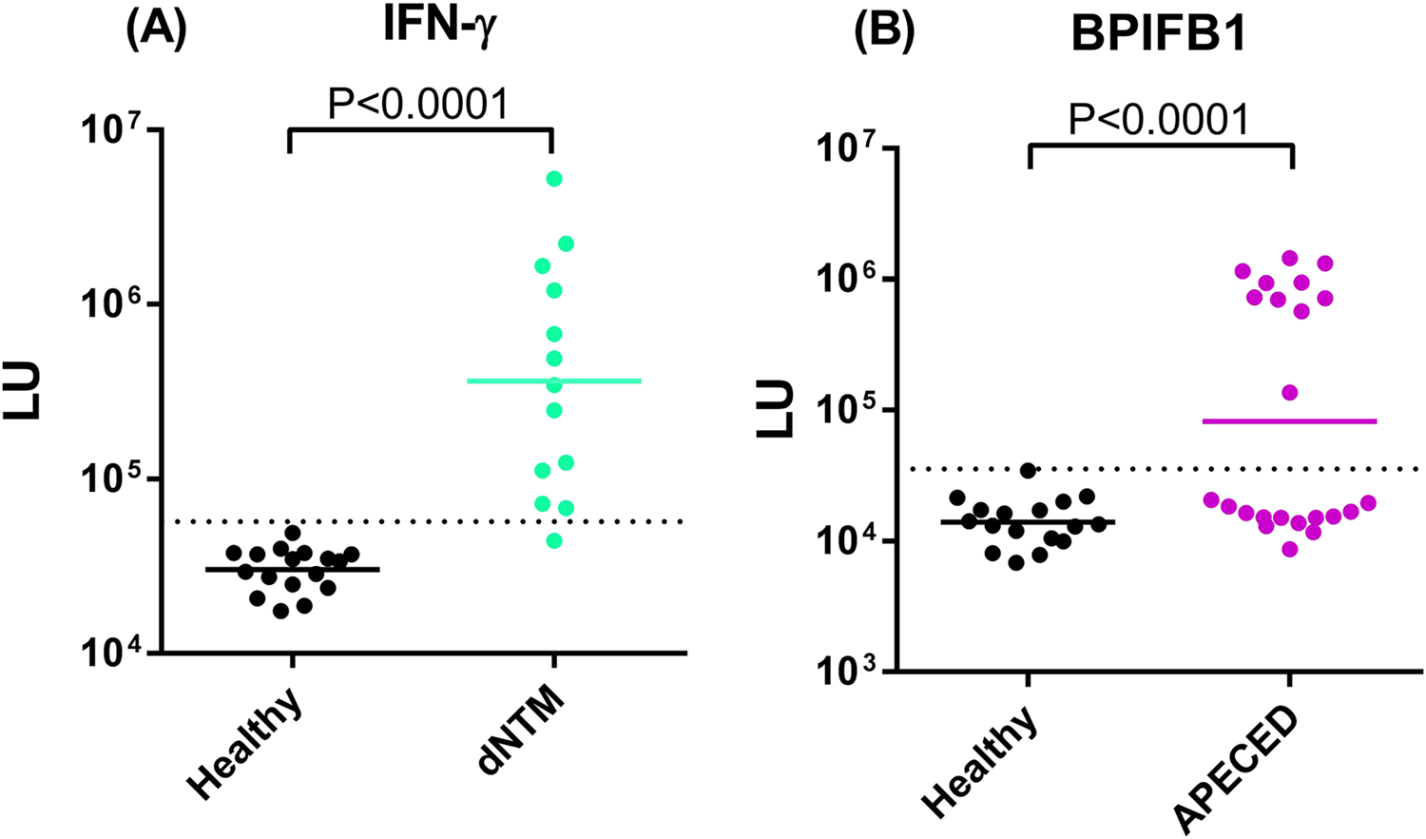
LIPSTICKS autoantibody detection for autoimmune disease diagnosis. (**A**) LIPSTICKS detection of serum autoantibodies against IFN-γ in a cohort of disease controls (black) and subjects with disseminated non-tuberculosis infection (eucalyptus green) demonstrated a diagnostic performance of 95% sensitivity and 100% specificity. (**B**) Autoantibodies against BPIFB1 in the sera of control subjects (black) and subjects with APECED (purple) were measured by LIPSTICKS. Only a subset of APECED patients had known BPIFB1 autoantibodies and comparison revealed that LIPSTICKS had a diagnostic performance of 95% sensitivity and 100% specificity.

Although autoantibodies represent key biomarkers for the diagnosis of most autoimmune diseases^23, 24^, few immunoassays are available for POCT. Autoantibodies can be directed against both intracellular and extracellular proteins, but autoantibodies targeting extracellular autoantigens, such as cytokines and receptors, can often directly cause pathogenesis^24^. To evaluate the utility of LIPSTICKS for detecting pathogenic autoantibodies against a cytokine, samples were analyzed from a previously characterized cohort harboring interferon-γ autoantibodies exhibiting an acquired immune condition often presenting as disseminated non-tuberculous mycobacterial (d-NTM) infection^25^. LIPSTICKS testing with a *Renilla* luciferase- interferon-γ fusion protein showed that the geometric mean level of autoantibodies in the d-NTM clinical subjects was 365,000 LU (95% CI 80,230–1,852,000) and was 12-fold higher than found in the healthy controls with a value of 30,220 LU (95% CI 27,000–35,740). Compared to our previous results with the standard LIPS assay, LIPSTICKS with the assigned cutoff value showed seropositive interferon-γ autoantibodies in 12/13 patients (95% sensitivity) and in none of the healthy controls (0/17; 100% specificity) (Fig. 5A). Lastly, examination of the global index of diagnostic accuracy of this LIPSTICKS test using AUC analysis revealed near perfect performance (AUC = 0.99; *P* < 0.0001).

Autoimmune polyendocrinopathy-candidiasis-ectodermal dystrophy (APECED) is a rare primary immunodeficiency characterized by a variety of symptoms including chronic mucocutaneous candidiasis, and heterogeneous autoimmune attack on endocrine and non-endocrine tissues^26^. APECED patients harbor a variety of autoantibodies including against cytokines and proteins associated with autoimmune-damaged tissues. One important biomarker of interstitial lung disease in APECED patients is the detection of autoantibodies against BPIFB1, which was originally characterized by a radioactive fluid-phase immunoassay^27^. For LIPSTICKS, an N-terminal BPIFB1-*Gaussia* Iuciferase fusion protein was utilized for autoantibody detection. Testing of serum samples from a cohort of healthy controls and seronegative and seropositive BPIFB1 APECED patients revealed that the geometric mean antibody levels of the BPIFB1-seropositive samples were 52-fold higher than the seronegative samples (Fig. 5B). Since only a subset of the APECED subjects harbor BPIFB1 autoantibodies, we compared the results to the standard 2.5 h LIPS assay^28^ and found the two assays were highly concordant with LIPSTICKS showing 95% sensitivity and 100% specificity. Together these results with interferon-γ and BPIFB-1, highlight the high performance of LIPSTICKS for the ultrarapid detection of autoantibodies directed against extracellular targets associated with autoimmune conditions.

### Serological diagnosis of Sjögren’s syndrome by LIPSTICKS

**Figure 6:**
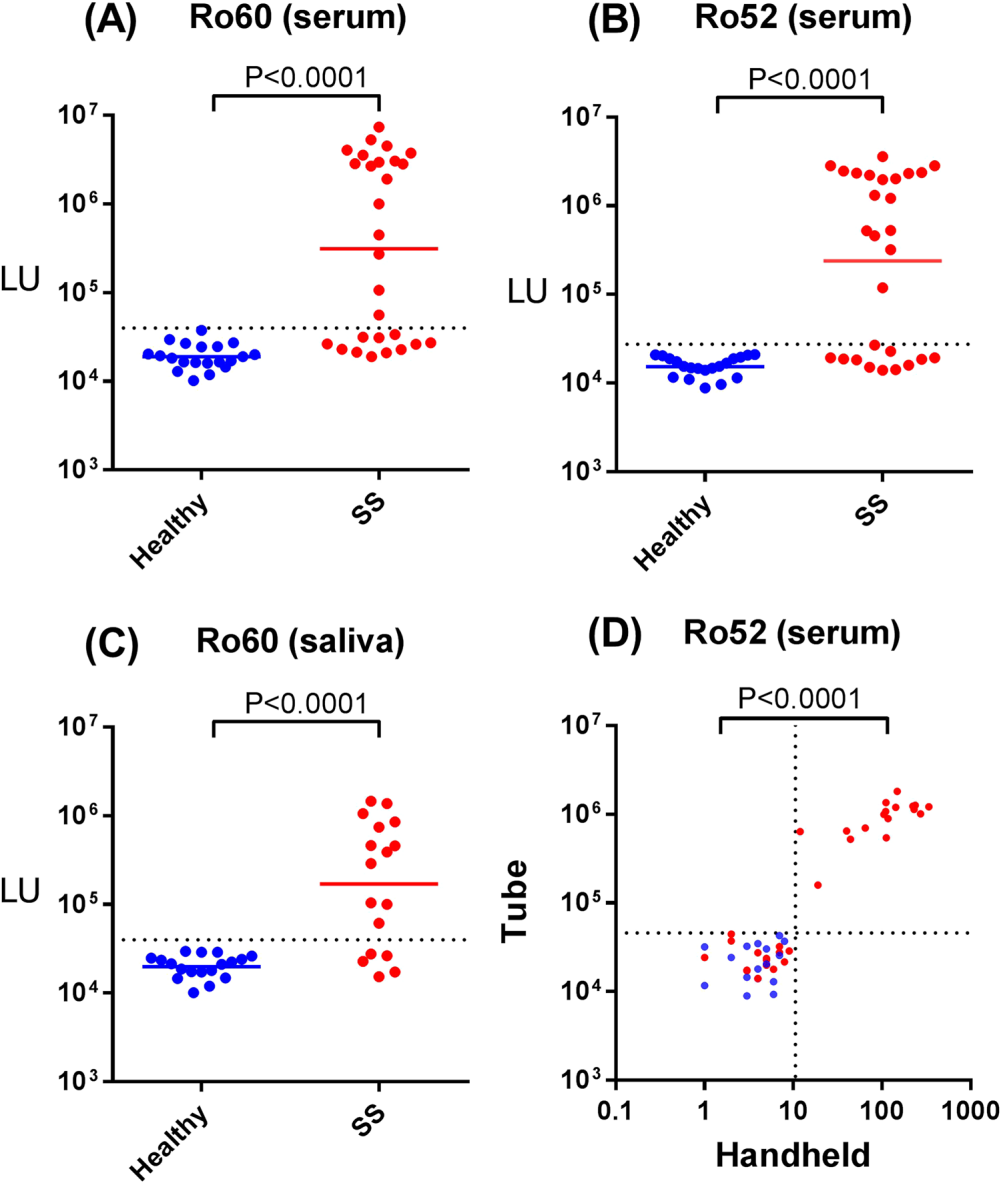
Ultrarapid diagnosis of Sjögren syndrome using serum and saliva. Serum autoantibodies against Ro60 (**A**) and Ro52 (**B**) were detected by LIPSTICKS using *Renilla* luciferase fusion proteins in healthy controls (blue) and subjects with Sjögren’s syndrome (red). (**C**) Autoantibodies were also detected directly in 10 μl of saliva (without dilution) by LIPSTICKS in healthy controls (blue) and subjects with Sjögren’s syndrome (red). These results with saliva matched the ELISA using the corresponding serum from these individuals. (**D**) Ro52-NanoLuc fusion protein was used to measure autoantibodies by LIPSTICKS in healthy controls (blue) and patients with Sjögren’s syndrome (red). Shown is the comparison of the detection of the luciferase–generated light with the standard tube luminometer vs. the hand-held luminometer. The correlation coefficient between the values obtained with the two devices was R = 0.73 (p < 0.0001).

Sjögren’s syndrome (SS) involves chronic inflammation and autoimmune attack on the salivary and lacrimal glands resulting in the loss of saliva and tear production, respectively^29^. One diagnostic criterion for diagnosis of SS is the presence of autoantibodies against SSA (Ro52 and Ro60) and SSB, which are present in approximately 70% and 40% of patients, respectively^30^. Although most SSA immunoassays detect autoantibodies against both Ro52 and Ro60, separate recombinant proteins can be used for testing. To determine the performance of LIPSTICKS for detecting autoantibodies for SS diagnosis, a cohort of serum samples from healthy controls (n = 20) and SS patients (n = 28) were evaluated by LIPSTICKS using *Renilla* luciferase-Ro60, *Renilla* luciferase-Ro52 and *Renilla* luciferase-La and the results were compared with clinical ELISA data. As shown in Fig. 6A, much higher LU values for Ro60 autoantibodies were observed in the SS cohort compared to the healthy controls, yielding 61% (17/28) sensitivity and 100% specificity. The results produced were identical with the conventional ELISA. Similar results were obtained with Ro52 (Fig 6B). In addition, the SSB/La antigen showed promising results by LIPSTICKS and showed 52% sensitivity and 100% specificity and even detected two additional positives missed by the ELISA (Supplemental Fig. 3).

### LIPSTICKS for salivary diagnosis of SS

Saliva represents a convenient biomarker fluid due to its simple and non-invasive collection^31^. We have previously demonstrated that 5 µL of undiluted saliva can be employed in LIPS to measure autoantibodies for the diagnosis of SS^32^. Due to the known lower levels of IgG antibody levels in saliva^33^, we tested whether 10 µL of saliva could be directly used without dilution in LIPSTICKS for SS diagnosis. For these studies, we used a cohort of saliva samples from healthy volunteers (n = 18) and SS patients (n = 17) and the LIPSTICKS results were compared to serological testing of these same subjects based on clinical diagnosis and SSA ELISA data measuring both Ro52 and Ro60 together. Similar to the serum based testing, the autoantibody levels found in the SS subjects were approximately 20 times higher than the healthy controls. Using the controls for determining the cut-off value revealed that the LIPSTICKS test with *Renilla* luciferase-Ro60 on saliva showed 70% sensitivity (100% specificity) for SS diagnosis (Fig. 6C) and showed identical results with the results from the serum SSA ELISA. The LIPSTICKS *Renilla* luciferase-Ro52 test on the same saliva samples was less informative than Ro60, with only 53% sensitivity (100% specificity) for SS diagnosis (Supplemental Fig. 4). Further analysis, revealed that the Ro60 saliva test had excellent diagnostic accuracy with AUC value of 0.87 (*P* = 0.002). Together these results, particularly with Ro60, demonstrate that saliva LIPSTICK testing can be used to efficiently diagnose SS.

### Application of a hand-held luminometer for LIPSTICKS testing

To our knowledge, only table-top luminometers with photomultiplier tubes that require high-voltage power sources have been employed in clinical diagnostics based on luciferase activity. However, handheld, battery-operated luminometers with photodiode detectors are routinely used for ATP/luciferase-based testing of bacterial contamination^34^. Here, we explored whether a portable hand-held luminometer could be combined with LIPSTICKS for mobile antibody testing. Initial calibration experiments of the light output generated by *Renilla* luciferase-antigen fusion proteins with coelenterazine (flash) or even a glow substrate was insufficiently weak to be detected by the photodiode luminometer. As an alternative reporter, we evaluated NanoLuc that is a luciferase which exhibits a sustained glow and has higher specific luciferase activity using a NanoGlow substrate^35^. We generated a Ro52-NanoLuc fusion construct that yielded approximately ten times more luciferase activity than the corresponding *Renilla* luciferase-Ro52 antigen. Additional experiments revealed the Ro52-NanoLuc extract was bright enough to be used with the handheld luminometer, but this instrument had a lower signal output range (i.e. 0–10,000 LU) compared to the tube luminometer (Supplemental Fig. 5). The Ro52-NanoLuc extract was employed in LIPSTICKS assay with the hand held luminometer to formally evaluate autoantibodies in the sera of healthy volunteers and SS patients. As shown in Fig. 6D, the antibody levels observed in the SS subjects (GML of 28.5 LU) were much higher than the healthy controls (GML of 3.8 LU). Employing the cut-off value based on the mean plus 3 SD of the controls showed a diagnostic performance of 65% sensitivity and 100% specificity and produced values that tracked the values obtained with the tube luminometer (Fig. 6D). Moreover, the diagnostic performance of LIPSTICKS with the handheld luminometer was equivalent to an ELISA requiring several hours to complete. These findings indicate that LIPSTICKS can be combined with a portable device to detect autoantibodies for the diagnosis of SS.

## Discussion

POCT is gaining increasing popularity due to its positive impact on improving health care decisions^36^. To date, POCT based on clinically useful antibodies has mainly involved the lateral flow immunoassay technology directed at the diagnosis of several infectious diseases (e.g. HIV). For the first time, we describe a fluid-phase immunoassay, LIPSTICKS, designed for ultrarapid POCT of both infectious and autoimmune diseases. We demonstrate its high diagnostic performance with respect to three infectious and three autoimmune diseases. Examination of the global index of diagnostic accuracy of the LIPSTICKS tests using area under the receiver operator curve (AUC) revealed they showed high performance (e.g. AUC = 0.99 for detecting IFN-γ autoantibodies and AUC = 0.87 for the Ro60 SS test). LIPSTICKS has several advantages over lateral flow immunoassays including fast reaction kinetics, the ability to efficiently detect conformational epitopes, quantitative results and large dynamic range of detection. LIPSTICKS requires only a limited number of simple steps involving the direct capture and processing of antibody-luciferase-labeled antigen complexes with the aid of paramagnetic protein A/G beads and neodymium magnets. The neodymium magnets are relatively inexpensive ($0.75) and can easily be cleaned and reused. Moreover, we estimate the cost for other reagents per LIPSTICKS test to be approximately $0.25.

One of the advantages of LIPSTICKS is that it only requires a small amount of sera (0.1 µL) or saliva (10 µL) per diagnostic test. A simple dilution step is required before testing sera, but saliva can be used directly without dilution simplifying POCT. Although saliva is known to contain secretory IgA immunoglobulin, it also contains large amounts of IgG derived from serum through gingival crevices and thus represents an accessible window into the systemic body^33^. Particularly encouraging is our successful detection of SS autoantibodies in patient saliva. Similarly, Tiberti *et al*. found with a radiobinding assay that saliva was diagnostically as informative as serum for detecting autoantibodies associated with T1D^37^. Based on these and other findings, it is likely that salivary antibody-based diagnostics could be developed for many other infectious and autoimmune diseases.

A key feature of LIPSTICKS technology is its modular format, in which serological tests can developed for different autoimmune or infectious disease by simply changing the luciferase-tagged antigen. Based on the successful utility of LIPS for the diagnosis of several domestic and global pathogenic agents including HCV^38^, Lyme disease^39^, *Strongyloide*s^40^, *Loa loa* ^41^, and *Onchocerciasis* ^42^, it is likely that LIPSTICKS could be developed for these and other pathogens. The ability of LIPSTICKS to efficiently detect autoantibodies against Ro52, Ro60 and La suggests potentially promising results for generating a diagnostic profile with systemic lupus erythematosus^43^ and other autoimmune conditions. Our finding that a portable, hand-held luminometer can be used to measure antibodies with LIPSTICKS offers a practical and cost-effective option for diagnosing disease and may even be useful for vaccine monitoring. In some cases, improved analytical sensitivity may be needed for detecting other diagnostic autoantibodies showing lower amounts and/or affinity. Additional optimization could increase the detection limits, including with improved blocking reagents for lowering the non-specific immunoreactivity, the generation of higher specific activity reporters or even multiplexing. LIPSTICKS could be further standardized by including known amounts of commercially available purified antibodies and then using these values as reference. Lastly, due to its simplicity, an automated and miniaturized high-throughput platform might be able to interrogate clinical samples for a wide range of infectious and autoimmune disease providing a novel avenue for personalized health monitoring.

## Methods

### Serum and saliva samples

In accordance with the human experimentation guidelines of the Department of Health and Human Services, control and patient serum and/or saliva samples were obtained from studies in the intramural research program of NIH (Bethesda, MD) and University of California under multiple IRB approved protocols. These studies were approved by the committee on Human Research of the NIH Institutional Review Board (IRB-09-I-0030, IRB-99-CC-0168, IRB-07-I-0033, IRB-11-I-0187, IRB-11-D-0172) and University of California, San Francisco (IRB-10-01330). Written informed consent was provided by each subject who participated.

In order to show the broad application of the LIPSTICKS technology, we chose to study light-emitting fusion proteins based on published LIPS tests for three infectious diseases (HIV^18, 44^, EBV^19, 45^ and NHPV^21^), as well as for autoantibodies found in three autoimmune conditions (dNTM^25^, APECED^28^ and SS^30^). Available sera and/or saliva samples chosen in an unbiased fashion from the five different human cohorts of infectious or autoimmune diseases were used and each cohort also contained its own control samples. For proof-of principal studies for whether LIPSTICKS could be used to detect diagnostic antibodies in animals, additional serum samples from uninfected and equine nonprimate hepacivirus virus-infected horses (NPHV)^21^ were tested. Consistent with the large dynamic range of detection, power analysis confirmed that the number of samples tested for each disease cohort were sufficient for detecting statistical significant diagnostic results. Below we provide additional details about the different cohorts of clinical samples used for testing.

#### HIV cohort

Serum samples from HIV uninfected (n = 24) and HIV-infected subjects (n = 24) were used. The median HIV viral load from the untreated HIV-infected subjects was 29,494 copies/ml (interquartile range of 8,814–79,989). Additional testing from eight HIV-infected subjects from before and after long term anti-retroviral treated were also used and have been previously described^18^.

#### EBV cohort

Human samples were serologically evaluated for EBV infection with an EBV VCA ELISA (Trinity Biotech). However, following LIPSTICKS analysis, two samples detected as positive by LIPSTICKS but negative by ELISA were further studied in detail and were confirmed to be seropositive for two additional EBV antigens (p18 and p24) consistent with published studies. In total 39 samples, 15 EBV uninfected and 24 EBV-infected sera were studied.

#### NPHV

A cohort of horses with and without NPHV infection has been previously described^21^. A subset of horse samples with (n = 4) and without (n = 7) NPHV infection were used in LIPSTICKS.

#### dNTM

Disease controls and subjects having dNTM with interferon-γ autoantibodies have been previously described^25^. For LIPSTICKS, a blood donor controls (n = 17) and dNTM patients (n = 13) was studied and the results compared to previous testing^25^.

#### APECED

Serum samples from healthy controls (n = 17) and APECED subjects (n = 23) were used. Since no available ELISA data was available for BPIFB1 autoantibodies, the 2.5 hour LIPS assay with a BPIFB1-*Gaussia* luciferase detector was used to assess seropositivity as described^28^ and the results compared with LIPSTICKS.

#### SS

Healthy controls and SS patients were studied, in which the diagnosis of SS fulfilled the revised European consensus criteria^46^. Autoantibodies against SSA and SSB were determined by ELISA in the Laboratory of Clinical Medicine, Clinical Center, NIH. Saliva samples obtained from the parotid gland were obtained from SS (n = 17) and healthy volunteers (n = 18). Another set of serum samples from SS (n = 29) and healthy volunteers (n = 19) were also studied.

### Plasmids and recombinant antigens

Details about the ten different luciferase-antigen fusion constructs used in our study is provided in Supplemental Table 3. Mammalian expression plasmids expressing *Renilla* luciferase light-emitting antigen fusions for HIV p24^18^, HIV reverse transcriptase^18^, EBV EBNA1^19^, NHPV helicase^21^, human Interferon-γ^25^, human Ro52^30^, human Ro60^32^ and human La^30^, have been previously described. A BPIFB1-Gaussia fusion protein was also used to assess^28^. Lastly, a new Ro52-NanoLuc construct was generated and the details of this plasmid will be provided upon request.

To prepare recombinant light emitting proteins, Cos-1 cells were grown in DMEM supplemented with 10% FBS, 1% glutamine and penicillin-streptomycin and seeded in 100 mm dishes the day before transfection. Transfection of each of the plasmids (2 μg) was performed with Fugene-6 (Promega) per the manufacturer’s instructions. Forty-eight hours after transfection, the plates were washed once with PBS, scraped in 0.2 ml Buffer A (20 mM Tris, pH 7.5, 150 mM NaCl, 5 mM MgCl_2_, 1% Triton X-100), the cells were collected and centrifuged twice at 13,000 × g for 4 min, then the supernatants were collected and used immediately. Alternatively, the extracts can be harvested in Buffer A containing protease inhibitors and 50% glycerol and then stored frozen at −80 °C. Total luciferase activity in 1 μl of each crude extract was measured by adding it directly to 100 μl of assay buffer and substrate mixture (*Renilla* Luciferase Reagent Kit, Promega or in the case of the Ro52-NanoLuc, the Nanoglow substrate, Promega) in a clear 1.5 ml microfuge tube, vortexing and immediately measuring in a luminometer (Turner design 20/20, Promega) for 1 s.

### Magnets and paramagnetic beads

Two sizes of neodymium magnetic cylinders (“sticks”; K&G Magnets) were initially tested: 1.59 mm diameter X 25.4 mm thick (Cat # D2X0; 1/8″ diameter X 1″ thick) and 1/16″ diameter X 25.4 mm thick (Cat # D1X0; 1/16″ diameter X 1″ thick). The 1/16″ magnets produced a better detectable light signal, which was likely due to less physical quench of the light. To simplify handling of the magnets during the assay, two different size magnets were combined, whereby the 1/16″ diameter magnet was stuck together with the 1/8 diameter such that 1/16″ diameter magnet side was utilized to capture the paramagnetic beads (see Supplemental Video 1). Following LIPSTICKS testing with sera or saliva samples, the neodymium magnets were reused after decontamination and stripping of the bound protein A/G beads. This was easily accomplished by first placing in 0.1% bleach disinfectant, physically removing the paramagnetic beads from the magnets with a paper towel, and then rinsing the cleaned magnets with water.

Three different sizes of paramagnetic protein A/G-coated beads were tested including 2.5 µ diameter (Product #88803; Thermo Scientific/Pierce® protein A/G magnetic beads), 1 µ diameter Hi–Sur Mag Protein A/G (Ocean Nanotech), and 500 nm diameter Supermag protein A/G beads (Ocean Nanotech). The two smaller beads of 1 µ and 500 nm diameters needed more time, approximately 15 and 45 s, respectively, to be captured by the magnets from the 100 µL reaction volumes. Thus, the Thermo Scientific/Pierce protein A/G magnetic beads were used exclusively in LIPSTICKS. Additional experiments titrating these beads (Fig. 2C) revealed that 5 µL of beads (diluted 1:5 in water from the stock) provided an adequate signal to noise output for the LIPSTICKS assay.

### LIPSTICKS tube assay

The LIPSTICKS tube assay involves several steps and can be performed in approximately 45 s, in part due to the tube luminometer requiring only a 1 s integration time to read the sample (Fig. 1 and Supplemental Video 1). To initiate the assay, 5 μl of the diluted serum sample (1:10 in buffer A) or 10 μl of undiluted saliva was added to 5 μl of the luciferase-tagged antigen in a 1.5 ml microfuge tube. Of note, for the different antigen fusions used in the assays, the total input activity was approximately 50–100 million LU/μl. Next, 100 μl buffer A was added and the reaction mixture was immediately vortexed for 2 s. Then, 5 μl diluted paramagnetic beads (1:5 in water) was pipetted into the reaction mix and the tube was tapped two times to evenly disperse the beads. The magnetic stick was then immersed into the tube containing the beads for 5 s. The magnet was removed and dipped twice in wash buffer. Lastly, the magnetic stick was placed in a tube, preloaded in the luminometer (Turner design 20/20) and containing 100 μl coelenterazine substrate, and then read for an integration time of 1 s.

### LIPSTICKS with a Handheld Luminometer

The portable, handheld EnSURE photodiode luminometer (Hygiena) was also employed for testing. In order configure the device for LIPSTICKS, the Ultrasnap cap tubes supplied by the manufacturer (Hygiena) were first emptied of their contents and then rinsed four times with distilled water. The cleaned, empty tubes were then refilled with 100 μl Nanoglow substrate (Promega). To quantify the light emitted by the antigen/antibody complex, magnets bound with immune complexes were simply dropped into the Ultrasnap tube containing Nanoglow substrate, recapped and placed in the EnSURE luminometer and measured with an integration time of 15 s. Unlike *Renilla* luciferase antigen fusions, the NanoLuc reporter produced a high output with a stable glow with its substrate and is highly detectable during the long integration time with the handheld luminometer. Due to additional integration time of 15 s needed for the handheld luminometer, these tests required 1 min for completion.

### Data and Statistical Analysis

GraphPad Prism 6 software (San Diego, CA) was used for data analysis and for generating many of the figures. For each LIPSTICKS test, a standard statistical approach, grounded on the method described^47^, was used for determining the cut-off values and was based on the sum of the mean plus 3 standard deviations of the matched control samples. *P* values were calculated for the LIPSTICKS tests using the non-parametric Mann-Whitney *U* test. Additional receiver operator characteristic (ROC) of the diagnostic tests were performed. ROC analysis emanates from signal detection theory for evaluating how well a signal is detected compared to noise^48^. A key feature of ROC analysis is how well true positive samples (sensitivity) can be distinguished from the negative samples (specificity) and is used to evaluate the cut-off threshold on the outcome of a diagnostic test. The area under the ROC curve (AUC), a global indicator of diagnostic accuracy^49, 50^, was used to evaluate the performance of the LIPSTICKS tests.

## Electronic supplementary material

LIPSTICKS Demonstration

Supplementary figures
