# Supplementary material for: Ultrarapid Measurement of Diagnostic Antibodies by Magnetic Capture of Immune Complexes
Source: Sci Rep. 2017 Jun 19;7:3818. doi: 10.1038/s41598-017-03786-7 (PMC5476601; doi:10.1038/s41598-017-03786-7)
Supplement: Supplementary file 2 — Supplementary figures [file 41598_2017_3786_MOESM2_ESM.pdf]

Ultrarapid Measurement of Diagnostic Antibodies by Magnetic Capture of Immune Complexes

Peter D. Burbelo[1], Sreenivasulu Gunti[2], Jason Keller[3#], Caryn G. Morse[4], Steven G. Deeks[5], Michail S. Lionakis[6], Amit Kapoor[7], Qingxue Li[8], Jeffrey I. Cohen[8], Abner L. Notkins[2], and Ilias Alevizos[9]

Legends:

Suppl. Fig. 1. Decrease in HIV anti-RT antibodies before and after ART by LIPSTICKS
LIPSTICKS detection of antibodies against HIV reverse transcriptase (RT) in HIV serum sample taken from before and after ART. HIV samples used were previously described in Burbelo et al., JID, 2014.

Suppl. Fig. 2. LIPSTICKS detection of antibodies against equine nonprimate hepacivirus virus. Shown are antibody levels against the capsid for NPHV in seronegative (blue circles) and seropositive horse serum samples (red circles). The cut-off value for seropositivity is shown by the dotted black line.

Suppl. Fig. 3. **LIPSTICKS La serum autoantibody detection.** Shown is the LIPSTICKS detection of La antibody levels in a cohort of normal volunteers (blue) and Sjögren syndrome patients (red). The geometri mean in each group is shown by the horizontal bars and the cut-off value for seropositivity is shown by the dotted black line.

Suppl. Fig. 4. LIPSTICKS Ro52 serum autoantibody detection. Antibody levels are shown for a cohort of normal volunteers (blue) and Sjögren syndrome patients (red). The geometric mean in each group is show by the horizontal bars and the cut-off value for seropositivity is shown by the dotted black line.

Suppl. Fig. 5. Comparison of the photomultipler tube luminometer with the handheld photodiode-based luminometer. Known amount of Ro52-nanoluciferase were measured with both instruments and the LU values plotted against each other for comparison.

Suppl. Table 1 Capture Time of Different Paramagnetic Protein A/G Beads with Neodynium Magnets
Suppl. Table 2 Select Sera Seroreactivity to Three different EBV antigen
Suppl. Table 3 Description of Luciferase-Antigen Fusions Used for LIPSTICKS

Suppl. Video: LIPSTICKS demonstration

Suppl. Fig. 1

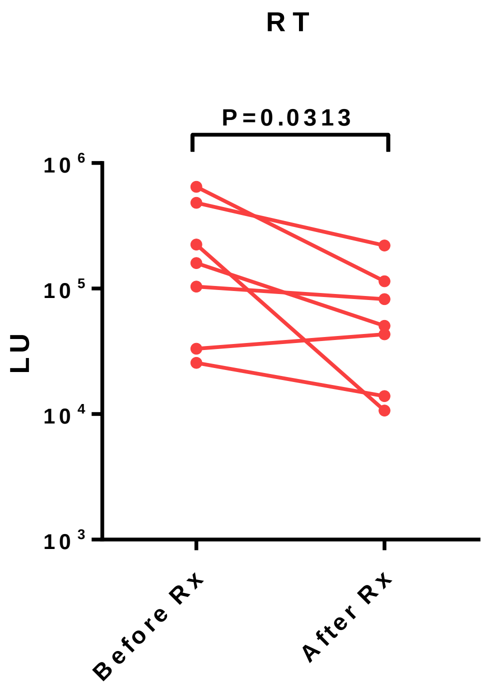

Suppl. Fig. 2

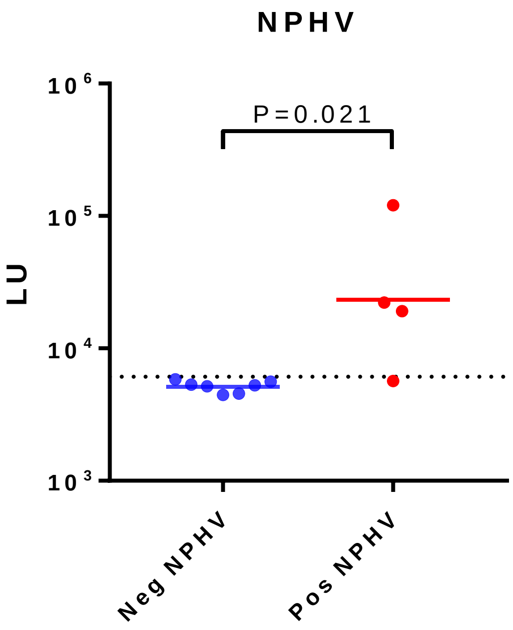

Suppl. Fig. 3

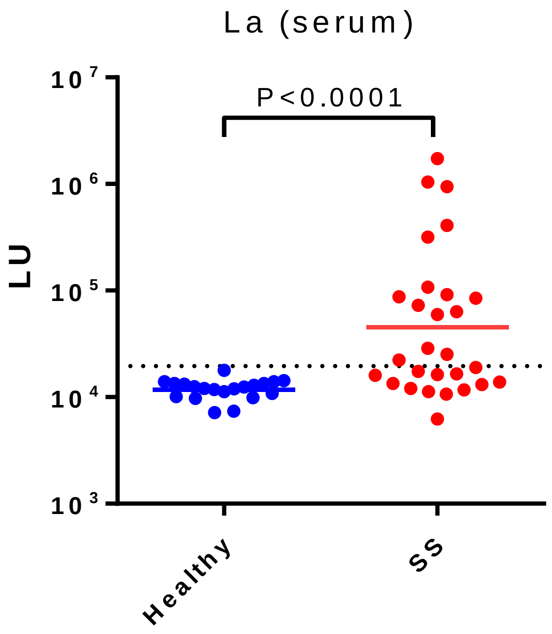

Suppl. Fig. 4

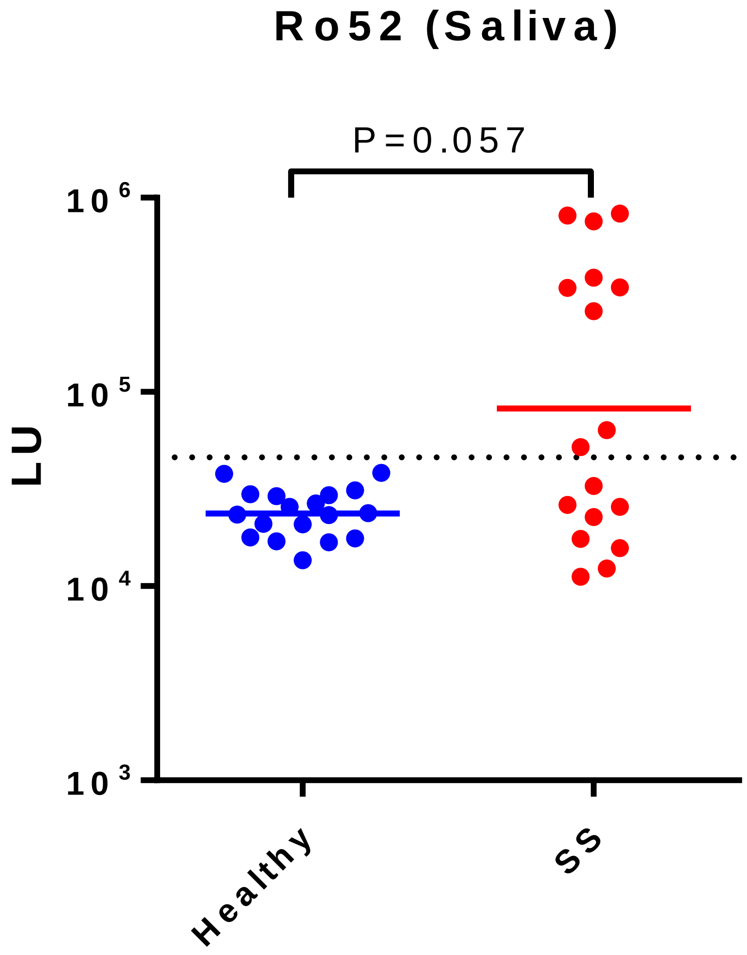

Suppl. Fig. 5

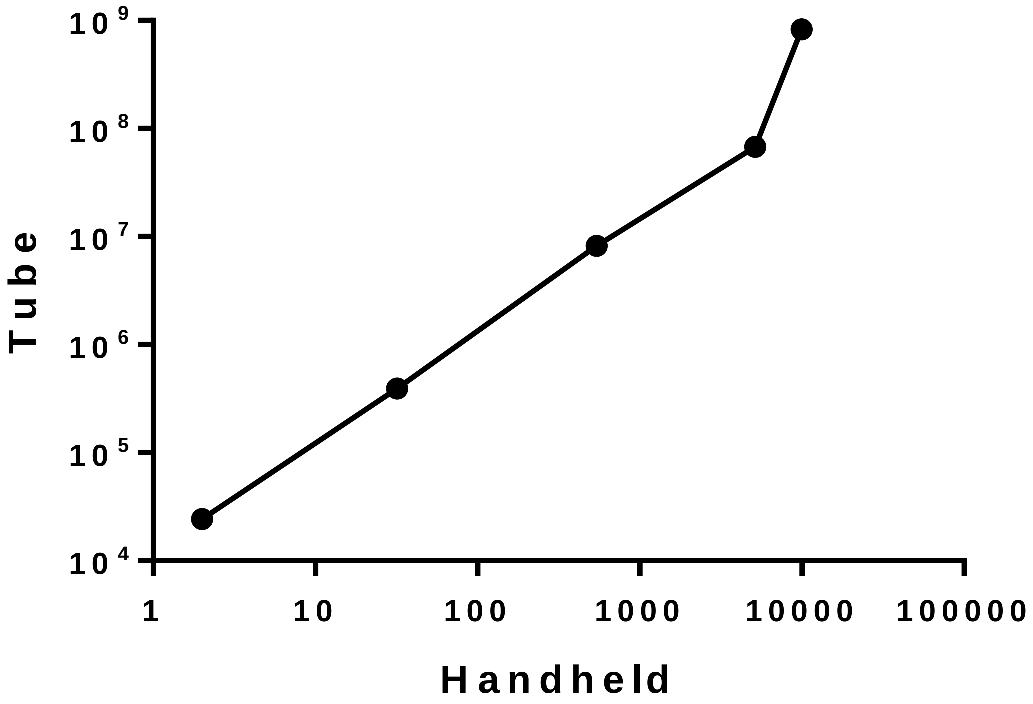

Tube
10^9
10^8
10^7
10^6
10^5
10^4
1
10
100
1000
10000
100000
Handheld

Supplemental Table 1 Capture Time of Different Paramagnetic Protein A/G Beads with Neodynium Magnets

Product Name	Vendor	Bead Size (diameter)	Approx. Time* (sec)
Pierce® Protein A/G beads	Thermo Scientific	2.5 μM	3-5
Hi–Sur Mag Protein A/G	Ocean Nanotech	1 μM	15
SuperMag Protein A/G	Ocean Nanotech	500 nM	45

* Capture time from 100 μl assay volume

Supplemental Table 2 Select Sera Seroreactivity to Three Different EBV Antigens*

SAMPLE	EBNA	GP42 (BZLF2)	BFRF-3 (CAPSID)
Buffer blank	neg	neg	neg
EBV neg by ELISA and neg by LIPS #90	neg	neg	neg
EBV neg by ELISA and neg by LIPS #85	neg	neg	neg
EBV pos by ELISA and pos by LIPS #29	Pos	Pos	Pos
EBV neg by ELISA and pos by LIPS #24	Pos	Pos	Pos
EBV neg by ELISA and pos by LIPS #15	Pos	Pos	neg

*Based on heterogeneous EBV humoral response profile seen in EBV-infected subjects as described in Bu et al. 2016, regular LIPS testing was performed was performed with three EBV antigens. The two samples #24 and #15 were positive for EBNA and gp42 and sample #24 was also seropositive with BFRF-3.

Supplemental Table 3 Description of Luciferase-Antigen Fusions Used for LIPSTICKS

Plasmid	Disease/ infection	Antigen	Luciferase	Type of antigen Fusion
pREN2-p24	HIV	P24 HIV capsid	*Renilla* Luciferase	C-terminal
pREN2-RT	HIV	P24 HIV reverse transcriptase	*Renilla* Luciferase	C-terminal
pREN2-EBNA1	EBV	EBNA1	*Renilla* Luciferase	C-terminal
pREN2-CHV	NHPV	Helicase	*Renilla* Luciferase	C-terminal
pREN2-IFN-γ	dNTM	interferon-γ	*Renilla* Luciferase	C-terminal
pGaus3-BPIFB1	APECED	BPIFB1	*Gaussia* Luciferase	N-terminal
pREN2-Ro52	SS	Ro52	*Renilla* Luciferase	C-terminal
pREN2-Ro60	SS	Ro60	*Renilla* Luciferase	C-terminal
pREN2-La	SS	La	*Renilla* Luciferase	C-terminal
pNano-Ro52	SS	Ro52	Nano luciferase	N-terminal
